# Assessment of antibacterial activity of self-etching dental adhesive systems: An *in vitro* study

**DOI:** 10.4103/0972-0707.48838

**Published:** 2008

**Authors:** Mithra N Hegde, Priyadarshini Hegde, Veena Shetty, Pavitra B Sampath

**Affiliations:** Department of Conservative Dentistry and Endodontics, A. B. Shetty Memorial Institute of Dental Sciences, India; 1 Department of Microbiology, K. S. Hegde Medical Academy, Mangalore - 575 018, India

**Keywords:** Adhesives, bacteria, dentine-bonding, direct contact test, microleakage

## Abstract

**Aim:**

To evaluate and compare the antibacterial activity of polymerized, two-step, self-etching and one-step, self-etching adhesive systems by using the direct contact test after one, seven, and fourteen days.

**Materials and Methods:**

The direct contact test was used to evaluate the antibacterial activity of Clearfil Protect Bond, Adper SE Plus, Clearfil SE Bond, and Adper Easy One on * Streptococcus mutans* after aging the samples in phosphate-buffered saline for one, seven, and fourteen days. Statistical analysis included the one-way Anova and Tukey's multiple comparison tests.

**Results:**

Among the tested materials, Clearfil Protect Bond exhibited an antibacterial effect for seven days when in contact with * S. mutans*. None of the adhesive systems exhibited any antibacterial effect after 14 days.

**Conclusion:**

The incorporation of antibacterial agents into dentine-bonding agents may become an essential factor in inhibiting residual bacteria in the cavity following a cavity disinfection procedure, and it could be recommended in situations where total disinfection of cavity is not accomplished due to lack of accessibility.

## INTRODUCTION

The development of adhesive systems that have enabled variable cavity design to preserve the intact tooth structure and treatment of caries, has recently been shifted from the traditional method to that with down-sized cavities. However, when attention is focused on decreased removal of the tooth structure; it is possible that some active bacteria reside in the cavity. On the other hand, the number of dentulous elderly patients is increasing, a trend that has increased the incidence of root caries. Many root surface, carious lesions are extensive and adequate caries removal and restoration placement is difficult.[1]

Dentine adhesives are currently available as three-step, two-step, and single-step systems depending on how the three cardinal steps of etching, priming, and bonding to the tooth substrate are accomplished or simplified. In the three-step system, etching, priming, and bonding are carried out in three different steps. Two-step systems are subdivided into self-priming adhesives that require a separate etching step, and self-etching primers that require an additional bonding step. The recently introduced, all-in-one adhesives have further combined these three bonding procedures into a single-step application.[2]

To provide resin-based materials with antibacterial activity, a new monomer, methacryloyloxydodecylpyridinium bromide (MDPB) with antibacterial activity against oral Streptococci has been developed, which is a quaternary ammonium compound with a methacryloyl group.

**Figure 1:**
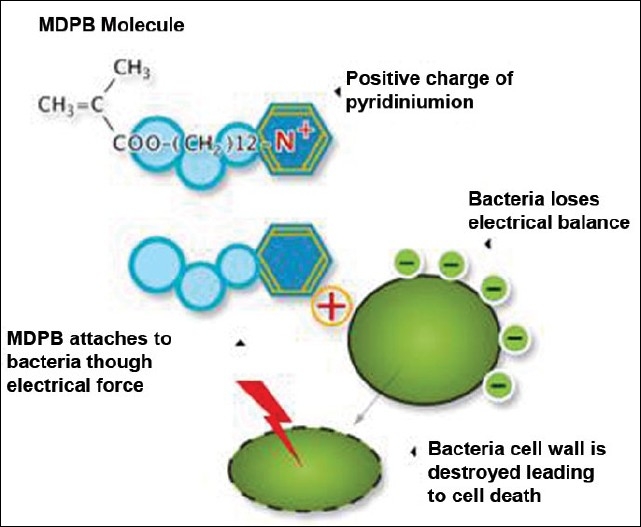
Structure of MDPB molecule and its action on bacterial cell

The pyridinium group as a component of the MDPB monomer (12-methacryloyloxydodecyl pyridinium bromide) is positively charged. The cell structures of bacteria are generally negatively charged and are automatically attracted by the positive charge of the MDPB monomer. They then lose their electrical balance, which destroys the cell membranes of the bacteria [Figure 1]. The bacteria are killed by this process known as bacteriolysis, which reduces the presence of residual bacteria in the prepared cavity, which would otherwise induce recurrent caries and damage to the pulp.

## MATERIALS AND METHODS

### Tested materials

Four dentine-bonding agents were tested: Clearfil SE bond (Kuraray), Adper Easyone (3M ESPE), Clearfil protect bond (Kuraray), and Adper SE plus (3M ESPE). All materials were handled and polymerized in strict compliance with the manufacturers' instructions.

### Test microorganism and growth conditions

*Streptococcus mutans*, the primary etiological agent for caries, is widely used to test the antibacterial activity of dentine-bonding agents. A clinical isolate of the *S. mutans* 27351M, which is naturally resistant to bacitracin, was grown aerobically from a frozen stock culture in Brain–Heart infusion (BHI) broth containing 8 *μ*g/mL bacitracin for 48 hr at 37°C before being applied to the samples according to the experimental design described below.

## EXPERIMENTAL DESIGN

### Direct contact test

**Figure 2:**
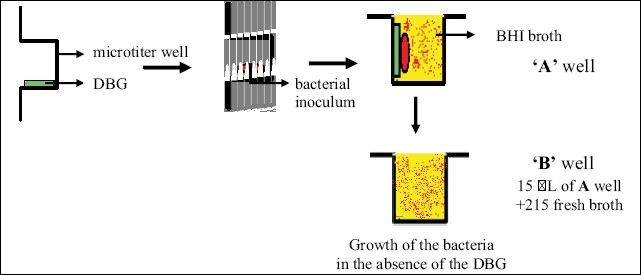
Schematic representation of direct contact test performed on 96 well microtiter plate. Bacteria are brought in direct contact with the tested material for 1hr followed by addition of growth medium

The Direct contact test (DCT) [Figure 2] is based on turbidometric determination of bacterial growth in 96-well microtiter plates. The kinetics of the outgrowth in each well is monitored at 600 nm at 37°C and recorded every 30 min using a temperature-controlled spectrophotometer.

### Schematic representation of the DCT

Three 96-well microtiter plates (288 wells) were divided into five groups: eight wells of each microtiter plate were utilized, of which four were designated as 'A' wells (with dentine-bonding agent and bacterial suspension) and the other four as 'B' wells (with the dentine-bonding agent but without bacteria which served as a negative control).

The dentine-bonding agents utilized in this study were divided into:

**Table:** 

Group 1	Group 2	Group 3	Group 4	Group 5
Clearfil SE bond	Adper Easy one	Clearfil protect bond	Adper SE plus	Control (bacteria but no dentine-bonding agent)

### Direct contact test

The 'A' wells were held vertically, *i.e*., the plate's surface was maintained perpendicular to the floor and the side walls of the wells were coated with 25 *μ*L of the respective dentine-bonding agent and cured. Special care was taken to avoid contact of the material with the bottom of the well, which would interfere with the path of light through the microplate well and result in a false reading.

Ten microliters of bacterial suspension (10^6^ cells) was placed on the test material. The plate was held in a vertical position and the wells were inspected for evaporation of the suspension liquid, which occurred within one hour at 37°C

This ensured direct contact between the bacteria and the tested material. Brain Heart Infusion broth (245 μL) was added to each of these A wells and gently mixed for two minutes.

Four B wells coated with the tested material but without any bacteria served as negative control whereas four wells (experimental A wells) with the bacterial broth but without the dentine-bonding agent served as positive control.

The plates were incubated at 37°C in the microplate reader and the optical densities were measured at 600 nm. The readings were taken immediately after polymerization, and after aging them in phosphate-buffered saline after one, seven, and fourteen days. During the aging process, phosphate-buffered saline was renewed every 24 hours. The results were statistically evaluated using the one-way Anova and Tukey's multiple comparison tests.

## RESULTS

Anova showed a significant difference in the bacterial growth rate, both on different adhesive materials (*P* < 0.001) and at different tested times (*P* < 0.001) [Table 1]. After the material had aged for one day, bacterial growth was observed in Clearfil SE bond, Adper easy one, and Adper SE PLUS and did not differ significantly from positive control growth [Table 2].

**Table 1:** Anova test between and within the groups

		Sum of squares	df	Mean square	F	Sig.
Baseline	Between groups	0.259	4	0.065	73.559	0.000
	Within groups	0.031	35	0.001	
	Total	0.290	39			
1^st^ day	Between groups	0.899	4	0.225	6.640	0.000
	Within groups	1.185	35	0.034		
	Total	2.084	39		
7^th^ day	Between groups	1.073	4	0.268	34.784	0.000
	Within groups	0.270	35	0.008		
	Total	1.343	39		
14^th^ day	Between groups	0.010	4	0.003	64.719	0.000
	Within groups	0.001	35	0.000		
	Total	0.012	39			

Sig.: Significance, p<0.001

**Table 2:** Bacterial growth in the five different groups after aging

	1st day	7th day	14th day
Group 1	0.57 ± 0.007	1.23 ± 0.05	2.19 ± 0.004
Group 2	0.68 ± 0.22	1.18 ± 0.07	2.18 ± 0.01
Group 3	0.66 ± 0.14	1.22 ± 0.13	2.15 ± 0.005
Group 4	0.20 ± 0.04	0.88 ± 0.06	2.15 ± 0.005
Group 5	0.77 ± 0.16	1.39 ± 0.1	2.17 ± 0.004

After aging for seven days, Clearfil protect bond still showed antibacterial activity although the other tested materials did not differ from the positive control [Table 2].

After aging for 14 days, none of the self-etching adhesives tested exhibited any antibacterial properties [Table 2].

## DISCUSSION

Microorganisms are directly associated with the etiology of enamel, dentine, and pulpal pathology due to caries. Secondary caries are the most common cause of dental restoration failure, presumably associated with residual bacteria and microleakage.[3]

The antibacterial activity of the self-etching primer tested was evaluated against *S. mutans*, the main pathogen responsible for the initiation of caries and the development of secondary caries.[3,4]

In self-etching adhesive systems, the pH of the self-etching primer solution is sufficiently low to demineralize the smear layer and the underlying dentine surface, so that etching and priming of the cavity can be accomplished simultaneously.[5] Therefore, the separate acid-etching step is generally omitted. Due to the nonrinsing procedure, residual bacteria may remain at the interface between the tooth and the restorative material. The dentine primer is the component that comes into contact and reacts with the dentin substrate at the first stage of restoration in an adhesive system. If tooth conditioners, such as primers, possessed antibacterial activity, these bacteria could be eliminated, thereby preventing secondary caries. Thus, the antibacterial activity of these adhesive systems primers that are directly applied to the dentin, plays an important role in the longevity of the restoration.[6]

The direct contact test employed in our study has many advantages over the agar diffusion test and has been studied previously by Weiss *et al.*, Shalhav *et al.*, and Fuss *et al*. It is a quantitative assay which allows water-insoluble materials to be tested. It relies on direct and close contact between the test microorganism and the tested materials and is virtually independent of the diffusion properties of both the tested material and the media. In addition to its reproducible and quantitative nature, DCT is relatively insensitive to the size of the inoculum brought in contact with the tested material. It thus facilitates simultaneous, standardized measurements of a large number of specimens and their respective controls on the same microtiter plate and has the ability to monitor the microbial growth, both in the presence and absence of the tested material.[7]

Clearfil protect bond showed antibacterial activity compared to the other self-etching primers tested, an effect that lasted for more than seven days. This is in agreement with the study done by Osnat *et al*. in 2007 who also found that the antibacterial effect of Clearfil protect bond lasted for seven days.[8] The reason for the prolonged antibacterial effect of Clearfil protect bond is related to the antibacterial properties of the MDPB molecule, which is a quaternary ammonium derivative synthesized by combining dodecylpyridinium bromide with a methacryloyl group.[9]

The mechanism of the antibacterial activity of quaternary ammonium compounds is believed to be due to their cationic and hydrophobic binding to cell wall components that disturbs membrane function and subsequently, induces leakage of cytoplasmic material.[10]

Clearfil SE bond does not show any antibacterial activity and it did not differ significantly from the positive control. This is in agreement with a study done by Juliana *et al*. in 2008 who found that light activation of Clearfil SE bond did not show any antibacterial activity.[11]

Adper SE plus is known to have a pH lower than 1 and Adper easy one has a pH of 3.5; neither shows any antibacterial activity. Our results are thus in agreement with the study done by Imazato *et al*. in 2003 who suggested that the benefit of the low pH environment exhibited by dentine bonding agents should be considered as being limited.[12]

Thus, the incorporation of antibacterial agents into dentine bonding agents may be helpful in complete elimination of the residual bacteria from the oral cavity.

## CONCLUSION

The incorporation of antibacterial agents into dentine bonding agents may become an essential factor in inhibiting residual bacteria in the oral cavity following a cavity disinfection procedure. The self-etching adhesive containing the MDPB molecule showed antibacterial activity for a week and could be recommended in situations where total disinfection of cavity is not accomplished due to lack of accessibility.
